# From Straight Lines to Curved Paths: Validity and Reliability of Linear Position Transducers to Assess Linear and Angular Motion

**DOI:** 10.3390/s25195987

**Published:** 2025-09-27

**Authors:** Tom Lecocq, Maxime Truchon, Nicolas Tordi, Arnaud Gouelle

**Affiliations:** 1 Laboratory Performance, Santé, Métrologie, Société (PSMS), Faculty of Sport Sciences, Université de Reims Champagne-Ardenne, 51 100 Reims, France; tomlecocq39@gmail.com (T.L.); maximetruchon081@gmail.com (M.T.); 2 Fédération Française de Gymnastique, 7 ter, cour des Petites-Écuries, 75 010 Paris, France; nicolas.tordi@ffgym.fr; 3 UMR RIGHT, Université Marie et Louis Pasteur, 8 rue du Dr JFX Girod, 25 020 Besançon, France

**Keywords:** linear position transducer, angular movement, reliability, validity

## Abstract

For Linear Position Transducers (LPTs) to represent an ideal tool for velocity-based training, it needs to be both valid and reliable. Multiple studies assessed the reliability of LPT yet wrongfully incorporated biological variability. Moreover, all studies investigating validity conclude a negative impact of horizontal displacement, therefore constraining LPT use to solely multi-joint movement. The objectives were to assess the validity and the reliability of (1) the Tendo Sport LPT in a linear setting presenting almost no biological variability, and (2) an equation allowing the analysis of angular movement. (1) A weight of 10 kg was dropped vertically 100 times and both time and position measurement from the LPT were compared to motion equation. (2) Angular movements were performed first with a bike wheel and then by a human shoulder. The angles estimated with the equation, from LPT output, were compared to the angle measured from 3D motion capture. In the linear settings, bias, ULOA and LLOA are, respectively, equal to −0.008 s, +0.012 s and −0.016 s if errors come solely from the time measurement and 0.011 m, 0.029 m and −0.025 m if errors come solely from the distance. It is likely that error could come from both the time and the distance measurements. In the angular settings, the bike wheel condition yields excellent reliability (ICC = 0.9999) and good validity (RMSD = 9.12°), while the shoulder condition yields high validity (RMSD = 2.49°). LPT can be used to investigate angular kinematics in certain conditions described in this article and yield valid, reliable results for angles below 120°.

## 1. Introduction

Velocity-based training has emerged during the last few decades as an innovative way of prescribing strength and conditioning sessions [1,2,3,4], compared to the traditional planification based on volume (sets X reps) and intensity (%1RM, i.e., rep max). Its premises are simple; athletes should always move the barbell with the intention of having the highest possible velocity [5,6,7]. Its advantages are multiple: self-regulation of the training session (load, sets, reps) thanks to real-time feedback regarding the attained velocity during the concentric part of the movement, sub-maximal 1RM estimation related to the relationship between load and velocity [2] and athlete’s readiness and level of fatigue monitoring. The velocity of the barbell is the most determinant factor in velocity-based training; therefore, it needs to be accurately measured.

**Figure 1:**
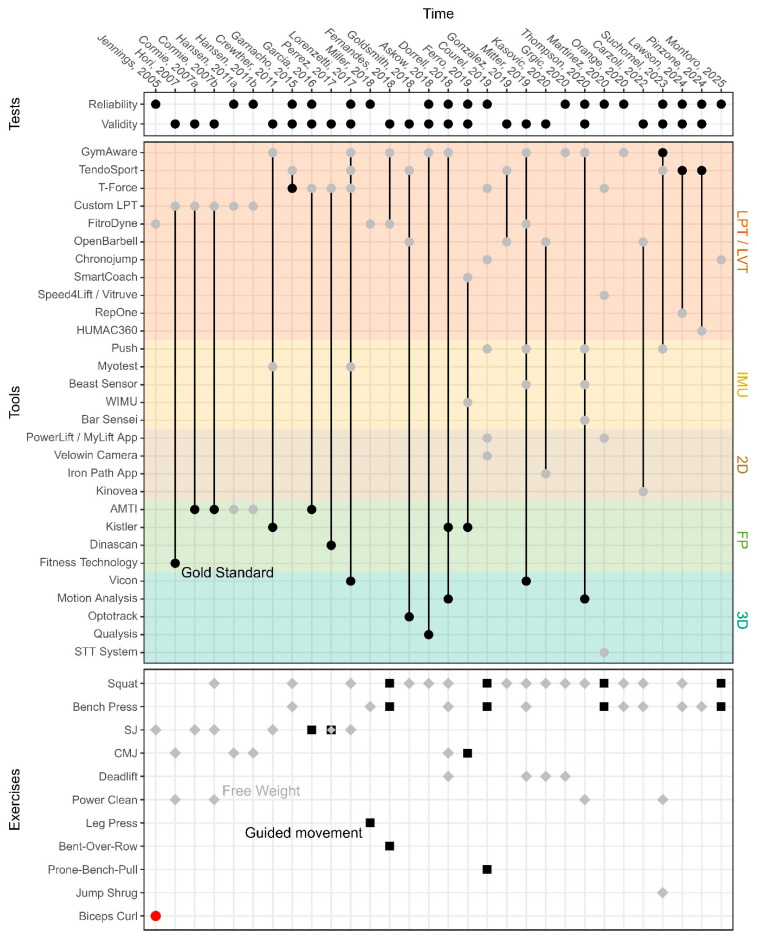
Illustration of the technologies and exercises selected in articles investigating the validity and reliability of Linear Position Transducers/Linear Velocity Transducers, published between 2005 and 2025. Facet 1 (Tests): whether the article investigates reliability, validity or both. Facet 2 (Tools): investigated equipment as gray point and gold standards as black point. Facet 3 (Exercises): free weight exercise as gray diamonds, guided machine as black squares and single joint exercise as red dot. LPT: Linear Position Transducers. LVT: Linear Velocity Transducers. IMU: Inertial Measurement Unit. 2D: Camera. FP: Force plates. 3D: Motion Capture. SJ: Squat Jump. CMJ: Counter Movement Jump.

Multiple tools and methods have been developed and used throughout the last years to measure the barbell’s velocity (see Figure 1 for an overview of tools and methods investigated). However, sessions and subsequent performances can be strongly affected by the reliability and the validity of the different tools used during training that can positively or negatively impact the lives of athletes and coaches [8].

The velocity of an object can be measured directly with a sensor or computed indirectly from two different branches of dynamics. On the one hand, kinetics focuses on the forces that cause motion. It is possible to compute the velocity from the force–time data thanks to the impulse–momentum relationship. On the other hand, kinematics focuses on motion without considering the causes. Position–time data provide velocity– and acceleration–time data after first and second differentiation, respectively. Once the basic kinematic variables are uncovered, the force generated can be computed by factoring the mass displaced into the acceleration–time data. Similarly, power can be calculated by factoring the force into the velocity–time data.

Force plates have been used in sports science for kinetic measurements such as force and power. However, this technology might not be the best tool for velocity-based training. Indeed, the computed velocity corresponds to the system’s center of gravity (i.e., barbel + body) instead of solely corresponding to the barbel velocity. Accordingly, Hori and colleagues showed that errors are smaller when the movement of the barbell’s center of gravity moves parallel to the system’s center of gravity, such as during a loaded squat jump, compared to a Hang Power Clean [9]. Additionally, Garcia and colleagues later showed that errors are smaller with heavier weight compared to lighter weight during squat jumps, due to a smaller distance between the system and the barbell’s center of gravity [4].

3D Motion Capture systems (3D-MOCAP), considered the gold standard for kinematics in sports science, can be used for the accurate computation of instantaneous positions in three-dimensions at a sampling rate usually greater than 100 Hz. However, these procedures are usually expensive and too complex to be deployed widely for field testing.

Inertial Measurement Units (IMUs) composed of accelerometers and gyroscopes have also been used numerously in sports science [7,8,10,11,12,13]. IMUs are light and small enough to be directly attached to the barbell or the forearm of the athletes, allowing easy and rapid measurements in training settings.

Recently, Linear Position Transducers (LPTs) have gained popularity for field testing. Indeed, they allow tracking for each repetition, the speed of the barbell and other important characteristics. The principle behind it is simple yet ingenious. A cord, usually ±2.5 m long to allow Olympic lifts as well as vertical jumps, is wrapped around a rotating spool. When the cable is pulled from the sensor, a linear distance can be computed based on the spool rotation and its diameter. Two different technologies exist: (1) a light source and a photo detector located on each side of a perforated disk rotating with the spool (rotary encoder). After calibration, two light impulses, corresponding to two following perforations in the disk, can be converted into a linear distance; or (2) a potentiometer with a varying output voltage depending on the current position of the spool [14]. LPT, therefore, collects both time and displacement. Publicly available systems usually provide key aggregated variables to users, such as mean or maximal value for velocity, acceleration, force and power. However, some tools allow researchers interested in the continuous measure to export raw data.

Investigations comparing LPT to various other tools, including the gold standard, highlighted some discrepancies. The major default, often evoked in the scientific literature, is its inability to account for horizontal movement. As stated by Cormie and colleagues: “If the bar were to move 10° from a direct position under the potentiometer, the calculated vertical velocity would be overestimated by 1.54%.” [15]. This result was later highlighted visually by Carzoli and colleagues [16] and can be explained by a measured linear distance greater than the vertical distance traveled by the bar. Such a conclusion raises two problems. First, according to Courel and colleagues [8], “*it has been shown that small changes in the velocity developed against some reference workloads are accompanied by critical improvements in the neuromuscular and functional performance of well-trained athletes. For instance, an increment in mean concentric velocity of just 0.07 to 0.10 m/s is associated with improvements of ~5% 1RM strength in main resistance exercises such as the bench press, full back squat and prone bench pull*” [2,17,18,19]. It is, therefore, very important to have accurate and reliable tools to quantify real improvement compared to noise due to sensors or methods. Second, during free weight exercise, it is known that the bar is far from following a linear trajectory either in weightlifting [20,21] or in powerlifting [22]. Multiple solutions have been implemented to fix this issue. The Tendo Sport unit (Tendo Sports Machines, Trencin, Slovak Republic) also exists in a weightlifting version, which allows the user to reorient the cord vertically (i.e., perfectly under the bar) while at the same time placing the sensor unit further away from heavy weight and bouncing bars. Multiple researchers used a Smith machine to constrain the movement to solely vertical motion, therefore preventing any anterior–posterior or medio-lateral movement [4,8,11,23,24,25]. Leg press on a machine is also another example of a movement constrained to solely one plane [26]. A team of researchers synchronously used two LPT to triangulate the position of the barbell and address potential horizontal displacement [15,27]. Finally, the GymAware LPT (Kinetic Performance Technology, Braddon, Australia) accounts for deviation from verticality by quantifying the cord angle throughout time.

Moreno-Villanueva and colleagues wrote a comprehensive systematic review about the validity and reliability of LPT and Linear Velocity Transducers [28]. Figure 1 illustrates the different tools and exercises used in various articles included in the systematic review as well as a few other articles cited in the present research. Four different articles appear in the validity section without gold standard [6,16,29,30]. Those four studies are interested in the agreement between multiple tools, but none of them considered the reference.

Since most of the six different kinds of synovial joints (i.e., ball and socket, hinge, pivot, condyloid, saddle, plane) generate angular movement of one bone relative to the other, LPT can theoretically be used solely for movement involving at least two joints concurrently. Nevertheless, strength and conditioning sessions regularly include single joint exercises, such as biceps curl, while in such settings, LPT output variables are deeply flawed due to the circular trajectory of the segments.

**Figure 2:**
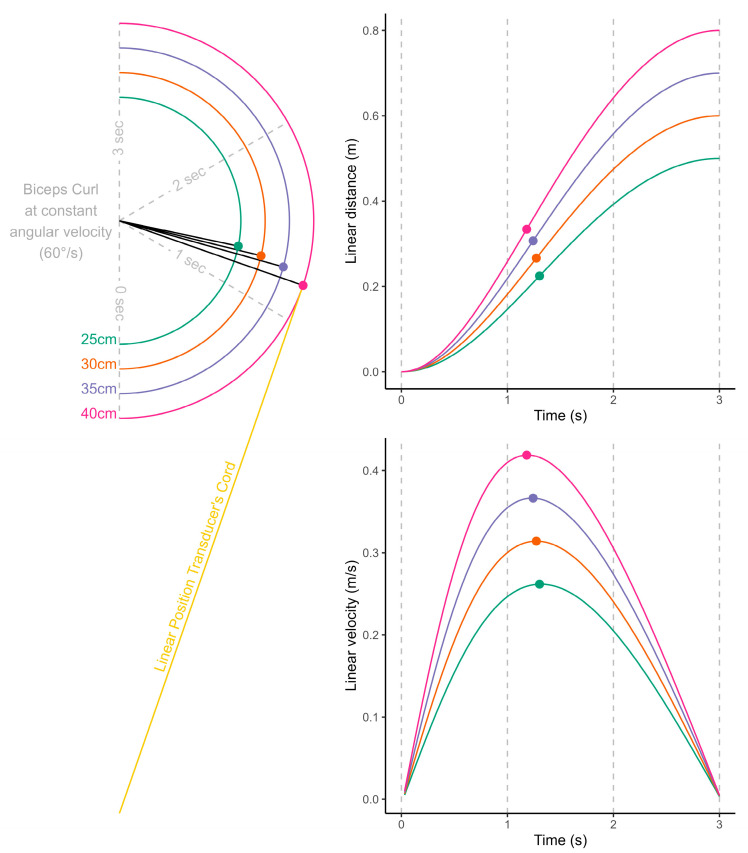
Illustration of Jenning’s article [1] shortcomings. The upper-body power estimated with LPT is unlikely to be accurate due to individuals having different forearm length (*N* = 30).

To the authors’ knowledge, only one article investigated such a movement. Jennings et al. concluded that muscle power can be measured with a high degree of reliability during a biceps curl thanks to the FitroDyne LPT [1]. However, due to the horizontal displacement of the hands, these results are highly likely to be biased, despite the following precaution taken by the authors: “*The FitroDyne device was placed on the floor in a position which allowed the extended nylon cord to be as close to the vertical plane as possible when the biceps muscles were contracted.*” [1]. As illustrated in Figure 2, even a movement performed at constant angular velocity would yield non-constant linear velocity with peak velocity measured when the forearms are perpendicular to the cord. Additionally, the horizontal displacement of the barbell (i.e., the length of the forearm) was different for every single recruited subject (*N* = 30; 24.8 ± 5.9 years; 178.8 ± 6.4 cm; 76.5 ± 11.0 kg).

Lastly, numerous articles assessed the validity and/or reliability of various LPT (Figure 1); however, as rightfully highlighted by Weakley and colleagues, “*studies that have quantified intradevice reliability have often failed to distinguish between technological and biological variability, which has likely altered the true precision of each device.*” [31]. Multiple articles falsely conclude about the sensors’ reliability while human beings performed the movement. While the question of human biological variability is crucial in sports science in order to distinguish any effect from noise (e.g., learning effects, training effects, fatigue effects, etc.), it should not be included in studies focusing on sensors’ reliability. Indeed, the sensor’s characteristics should stay true, independent of the tested population (e.g., training experience, number of familiarization sessions, etc.).

Therefore, the present article aims to assess the validity and the reliability of a widely used LPT in linear settings with a method that tends to reduce biological variability to a minimum and also in angular settings with both guided and free motion. It was hypothesized that (1) LPT could be more valid and reliable when assessed without biological variability and (2) they could be used to access angular motion under certain conditions, with improved validity and reliability for the guided motion.

## 2. Materials and Methods

### 2.1. Mathematical Foundations

**Figure 3:**
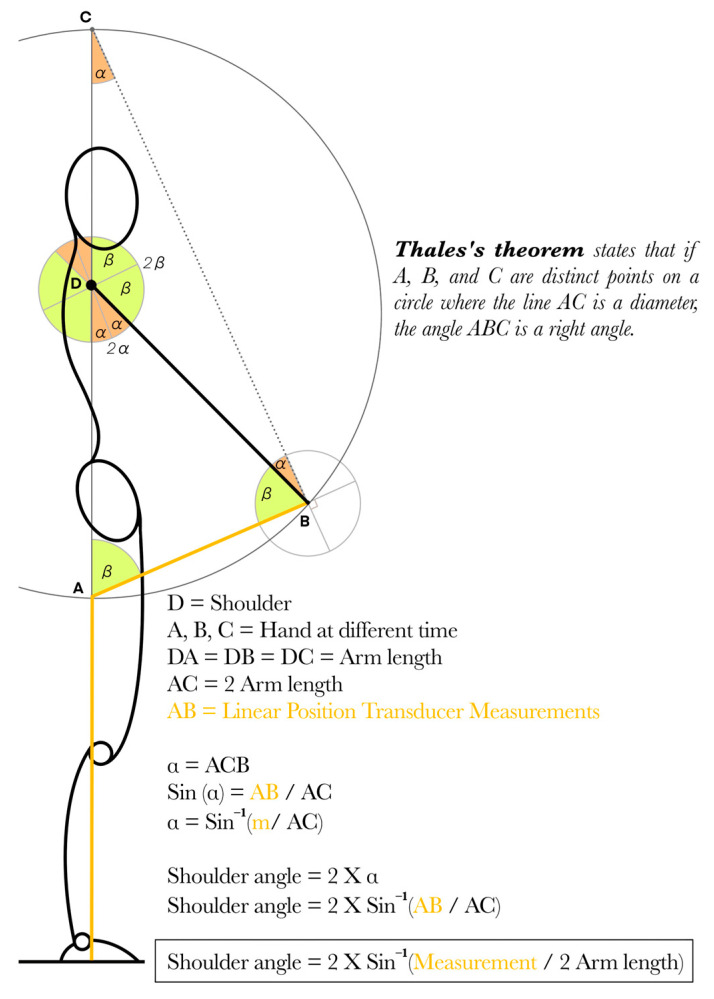
Presentation of Thales’s Theorem projected over a human movement as well as the formula allowing the computation of the shoulder angle at any given moment.

Thales’s theorem states that if A, B and C are distinct points on a circle where line AC is a diameter, the angle ABC is a right angle. During any human movement, shorter or longer segments revolve around the joint according to a circular path. However, if the length of the segment does not vary over time and the linear distance covered between the initial and the new position is known, then it is possible to triangulate the coordinate of the new position. By measuring the linear distance (circle’s arc) with an LPT, it should be possible to estimate the angle of the limb at any moment. The only restriction is to be placed in a Thales’s Circle; therefore, the linear distance of the new position needs to be computed from its initial position. This specification can be achieved by placing the LPT on the path of the segment, or more easily, by placing the LPT on the floor and redirecting its cord through the initial position of the limb with the help of a pulley, mounted on a tripod (Figure 3).

Before investigating angular kinematics in human beings (Part III), two other parts took place: The first part examined the validity and the reliability of an LPT in a standardized linear setting, with a phenomenon presenting no variability: vertical free fall under the universal law of gravity (Part I). Then the second part aims to retrieve angular kinematics with an LPT on a wheel. The wheel was chosen for its ability to reduce the number of degrees of freedom and constrain movement in a similar fashion to the use of a Smith machine during linear motion (Part II).

### 2.2. Part I—Free Fall Testing

The aim of the first part was to assess the validity and the reliability of the Tendo Sport LPT (Tendo Sports Machines, Trencin, Slovak Republic + Power Analyser Software (Version 7.1.4.0—2024)). The use of gravity as the only force acting was in agreement with the recommendation of Weakley: “*research assessing reliability of devices needs to account for, and preferably remove, biological variation to gain a true insight into a device’s reproducibility.*” [31].

A disk of 10 kg was dropped vertically 100 times from a standardized height of 34 cm above the floor and the LPT recorded its spatial and temporal parameters during the free fall. The Velcro strap of the Tendo Sport was attached to the weight. The Kevlar cord ran vertically above the disk and was reoriented toward the sensor unit with a pulley.

A height of 34 cm was chosen with the help of Equation (1), since from this height, the sensor measures a large range of potential human velocities.

(1) $$Velocity\;(m/s)=\sqrt{2\times g\times Height}$$

Indeed, human movement has been previously measured under 0.5 m/s with a load close to 1RM [2] and up to 2.5 m/s during ballistic movements such as squat jump [4].

A free body diagram of the fallen disk would present the force of gravity pulling the weight downward as well as two smaller forces going upward. In the first place, the default tension measured in the Kevlar cord of the Tendo Sport was about 1.5 N (150 g). This measure is coherent with a previous article: “*Cord tension of <200 g […] for the FitroDyne*” [29] knowing that “*FitroDyne and TendoSports Linear Position Transducer share a common background of technical design and development, but uses autonomous software for signal processing and data analysis*” [12]. In the second place, the drag force acting upward on the disk falling can be computed with Equation (2).

(2) $$Drag\;force\left(N\right)=\frac{1}{2}\rho SCV^{2}$$

The largest drag force computed with *ρ* the volumetric mass density of air (1.2 kg/m^3^), where *S* is the cross-sectional area of the body (0.64 m^2^), *C* is the drag coefficient (1) and *V* is the maximal velocity just before impact (2.5 m/s), is equal to 2.4 N. The cumulated forces heading upward are equal to 3.9 N or 3.9% of the weight just before impact and are consequently unimportant enough to be negligible in the rest of the article.

The spatial and temporal parameters measured by the Tendo Sport were, therefore, compared to motion equations (Equations (3)–(5)) of a free fall to assess the validity of the Tendo Sport.

(3) $$Acceleration\;(m/s^{-2})=g$$

(4) Velocity (m/s)=g×Time

(5) $$Position\;\left(m\right)=\frac{1}{2}\times g\times {Time}^{2}\;or\;Time\left(s\right)=\sqrt{\frac{2\times Position}{g}}$$

### 2.3. Part II—Ex Vivo Testing with a Wheel

The second part focuses on the validity and the reliability of the use of an LPT to retrieve angular kinematics of a bike wheel rotating thanks to an exenterated weight (Figure 3). Analogously to the use of a Smith machine to constrain a linear movement [4,8,11,23,24,25], the bike’s wheel was used to reduce the number of potential degrees of freedom in an angular movement.

**Figure 4:**
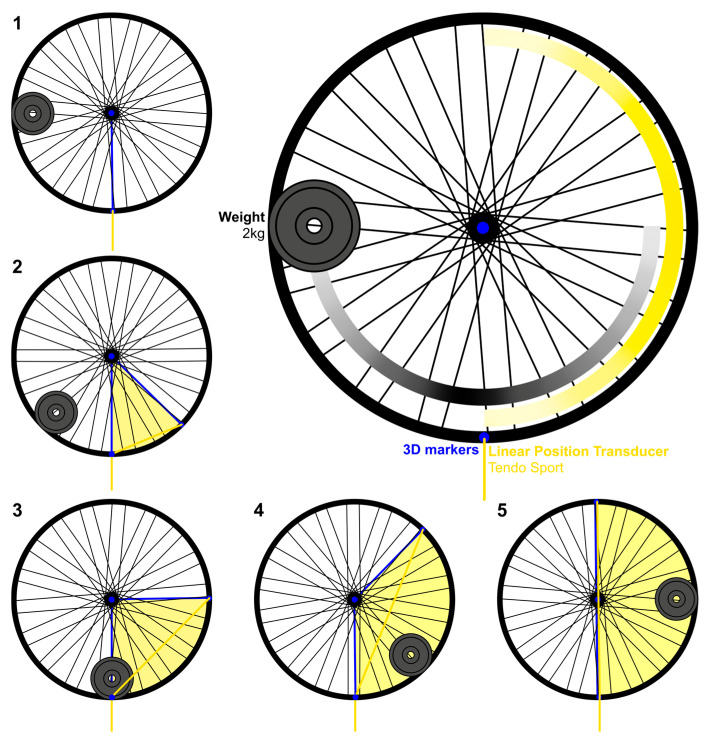
The wheel protocol allows comparison of the angle computed from the length of the Tendo Sport’s cord (yellow) to the angle computed with 3D-MOCAP (blue). The exenterated weight generates progressive angular acceleration and deceleration of the bike’s wheel, highlighted in sequential drawings from 1 to 5.

The Tendo Sport was tethered to the wheel thanks to the Velcro at the extremities of the cord. Additionally, the cable was held at the original position thanks to a pulley mounted on a tripod. The rotation of the wheel was standardized with the help of a 2 kg weight attached to the wheel with multiple plastic Rilsan collars. The weight was pulled back and up at a standardized height and dropped 100 times. The use of the upside-down front wheel of a bike with an exenterated weight is interesting, since it mimics the motion of the human body. After being released, the weight creates progressive acceleration during the first half of the movement and a progressive deceleration during the second half (Figure 4).

The movement was synchronously recorded by 3D-MOCAP. The protocol took place in a laboratory room surrounded by 10 cameras Flex 13 (OptiTrack, Corvallis, OR, USA) resolution 0.3 MP—640 × 480, sampling 120 Hz). Four reflexive markers (diameter = 14 mm) were attached to the bike wheel with double side tape. Two markers were placed on each side of the axis of the wheel and allowed the computation of the center of the axis of rotation. Two markers were attached on the opposite side of the wheel rim, where the Tendo extremity was tethered and allowed the computation of a theoretical point in the middle. The cameras’ position prevented occultation, and all markers were seen by at least two cameras at each instant to allow accurate reproduction. Potential optical interferences (e.g., light reflection on the bike frame) were masked with black matte tape. The calibration file with the manufacturer wand provided the following characteristics: overall reprojection average 3D error: 0.942 mm.

### 2.4. Part III—In Vivo Tests During Shoulder Flexion

Finally, the third part focused on the validity of the use of an LPT to retrieve angular kinematics from a human being. One of the authors volunteered to participate in this technical validation. After a free warm-up of 10 min consisting of upper-body mobility training, a single subject (28 years old, 1.83 m, 85 kg) performed 100 shoulder flexions at different speeds while holding a wooden stick with both hands. The subject was directed to perform 100 shoulder flexions at diverse velocities while keeping his arms extended and his scapula fixated throughout the range of motion. The movement was measured by both Tendo Sport, tethered to the wooden stick, and 3D-MOCAP. The protocol took place in the same laboratory and under the same calibration characteristics as part II. The anthropometric model is based on the work previously published [32]. Specific modifications were made to the models to meet the particularities of the project and the practical constraints of the experimental setup involving markers [33]. A total of 25 markers (Table 1) were attached to the upper body of the subject with double-sided tape. Joints’ center can be approximated as the midpoint between two anatomical markers. Such approaches are sometimes referred to as visual estimation methods and remain among the most frequently used. The center of the wrist was computed as the midpoint between the lateral and the medial marker placed on the wrist of the left arm. The center of the left shoulder was computed according to De Leva’s recommendation [34].

### 2.5. Data Analysis and Statistics

Data processing and data visualization were performed on R (version 4.4.0/2024-04-24) with RStudio (IDE version 2025.05.0) thanks to the Tidyverse library [35]. All markers’ coordinates were filtered with a Butterworth filter (order = 4, cut off frequency = 6) [33]. From the 3D-MOCAP data, the angles covered by the wheel and the shoulder were computed as the angle between the original position, the center of the axis of rotation and the new position. From the linear displacement measured by the Tendo Sport, the angle covered was computed with the help of the equation on Figure 3. As explained before, the Tendo Sport does not have an inherent sampling frequency; therefore, from the raw signals, both signals were interpolated at a frequency of 100 Hz to allow curve comparison. Once interpolated, all signals were synchronized at 10°; 0.25 s.

Part I allows the investigation of the reliability and the validity of the sensors. Reliability was assessed between the 100 identical trials, while validity was assessed against the theoretical motion equation. Part II also allows for the investigation of the reliability and validity of the new methods proposed in the present article. Reliability was assessed between the 100 identical trials, while validity was assessed against 3D-MOCAP. Finally, part III allows the investigation of the validity of the new methods on a human being against 3D-MOCAP.

Multiple descriptive statistics were computed to assess the validity and the reliability of each trial measured with the Tendo Sport device. Reliability was assessed with Intraclass Correlation Coefficient (ICC—two-way mixed effects, absolute agreement, multiple raters/measurements) between each trial and the average value of all trials. Validity was assessed with the Root Mean Square Differences (RMSD) between each trial and the gold standard. Both ICC and RMSD were computed individually for each trial. Minimal and maximal values were provided to show the range, while the average value gives an overview of the multiple measurements. The ICC was assessed against the following guidelines: values less than 0.5 are indicative of poor reliability, values between 0.5 and 0.75 indicate moderate reliability, values between 0.75 and 0.9 indicate good reliability, and values greater than 0.90 indicate excellent reliability [36]. The following statement of Song and colleagues about the lack of consensus are still relevant today and, therefore, the same subjective threshold from this single recent study was used to assess the RMSD: “*In absence of a research community consensus, we defined that the between-system magnitude difference is minimal if RMSD ≤ 5° for joint angles*” [33]. Finally, the Limits of Agreements (LoA) illustrated on Bland–Altman Plot allow for a visual and numeric assessment of the agreement between two methods. The agreement illustrates both the accuracy as well as the precision of the new method [37]. The range between the LoA (systematic error ± 1.96 random error) contains 95% of the paired differences solely if the sample follows a normal distribution [38]. Due to deviation from the normality of the residuals, non-parametric LoA were computed. The bias (systematic error) corresponds to the median of the paired differences, while the 2.5th and 97.5th quantiles represent, respectively, the lower and upper LoA (random error) [39]. Consequently, the systematic error sometimes does not lay perfectly in the middle of the LoA. With an equivalent 5° criterion as the clinically acceptable differences to assess the agreement, a systematic error smaller than 5° as well as an interval between LoA smaller than 10° would deem acceptable agreement between methods. Due to the squared function in the RMSD formula, the result can yield a solely positive value; therefore, the 10° range accounts for both 5° angle over- and underestimation.

## 3. Results

**Figure 5:**
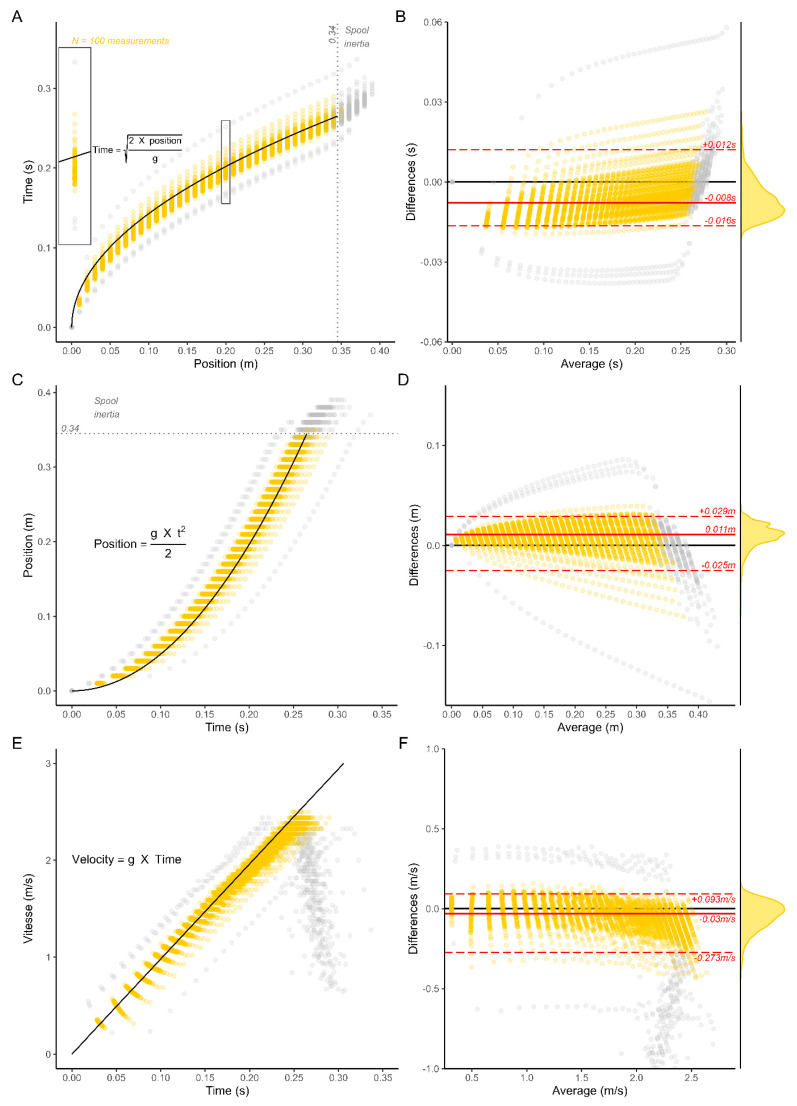
Validity and reliability of Tendo Sport LPT during the free fall of a 10 kg disk. Time (**A**), distance (**C**) and velocity (**E**) with matching Bland–Altman plot (**B**,**D**,**F**). As opposed to yellow points, gray points are displayed on the charts but removed from further analysis.

The results of **part I** are displayed in Figure 5. For each chart on the left, the 100 identical free fall trials measured with the Tendo Sport are represented against the corresponding motion equation represented by a black line. The points are slightly transparent to better highlight multiple overlays. The points presented in gray have been removed from the Bland–Altman analysis. The dropping height was 0.34 m; therefore, all longer lengths are incoherent and could be attributed to the inertia of the spool. Additionally, the 100 points at (0, 0) have also been removed, since these values are not measured by the sensor but artificially added afterwards as initial values. These 100 values standing perfectly on the theoretical model would have artificially improved the validity and the reliability of the tool. Additionally, after visual inspection, four curves were removed from further analysis. Their average z-scores were 5.40, −2.60, −2.81 and −3.06, confirming their outlier’s position. The Tendo Sport measures both time as well as distance to later compute velocity and other relevant training variables. Error could, therefore, potentially come from both time and/or distance. On the one hand, assuming that the position is accurate, Figure 5A represents time as the dependent variable and position as the independent variable. Based on this assumption, 95% of the paired differences between time measurements and motion equations are within −0.016 s and +0.012 s, with a systematic error of −0.008 s (Figure 5B). On the other hand, assuming that the time is accurate, Figure 5C represents position as the dependent variable and time as the independent variable. In this scenario, 95% of the residuals are between −0.025 m and 0.029 m with a systematic error of 0.011 m (Figure 5C). It is likely that error could come from both the time and the distance measurements. Finally, Figure 5E represents velocity as a function of time, computed from the raw measure of time and displacement. 95% of the residuals are between −0.273 m/s and 0.093 m/s with a systematic error of −0.03 m/s (Figure 5F).

**Figure 6:**
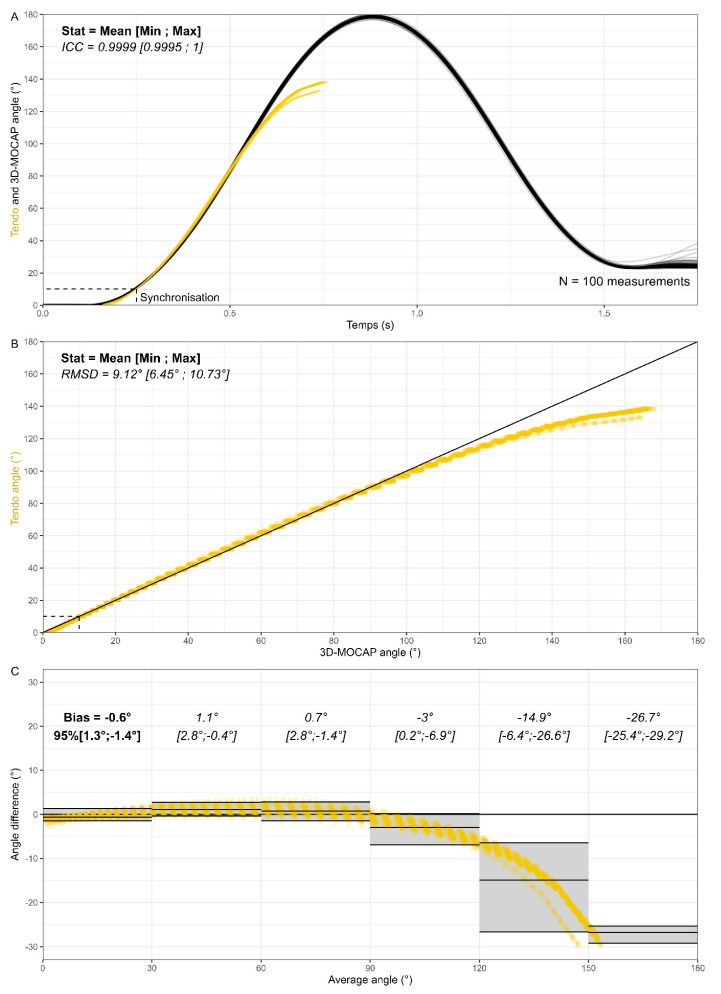
Validity and reliability of the Tendo Sport LPT to assess angular motion in the bike wheel protocol. Angle comparison (**A**), angle correlation (**B**) and Bland–Altman plot divided into meaningful sections (**C**). 3D-MOCAP in black and Tendo Sport in yellow.

The results of **part II** obtained with the bike wheel are displayed in Figure 6. The 100 releases are displayed as slightly transparent black and gold lines on Figure 6A and the perfect overlay suggests strong reliability. The black lines correspond to the angle obtained with the 3D-MOCAP while the gold lines correspond to the angle computed from the distance–time data of the Tendo Sport. Figure 6B presents the correlation of the angle measured with the two different tools. The gold standard (3D-MOCAP) is the independent axis, while the Tendo Sport is the dependent axis. The black diagonal represents the identity line (y = x). All points below the identity line highlight underestimation of the angle computed from Tendo Sport whereas all points above the line highlight overestimation. Finally, Figure 6C illustrates bias, as well as the upper and lower limits of agreements sectioned according to meaningful angular portions.

Strong reliability suggested by the clear overlay of the curves is numerically confirmed. The average value of the ICC for the 100 curve is 0.9999 with ICC = 0.9995 for the curve the farthest away from the average and ICC = 1 for the curve the closest to the average value. However, validity presents weaker results. The average value of the RMSD is 9.12° with individual curves within 6.45° and 10.73°. The individual values obtained are all above the threshold value of 5° criteria; however, it is very likely that large RMSD are strongly influenced by the right part of the chart (i.e., measuring large angles).

**Figure 7:**
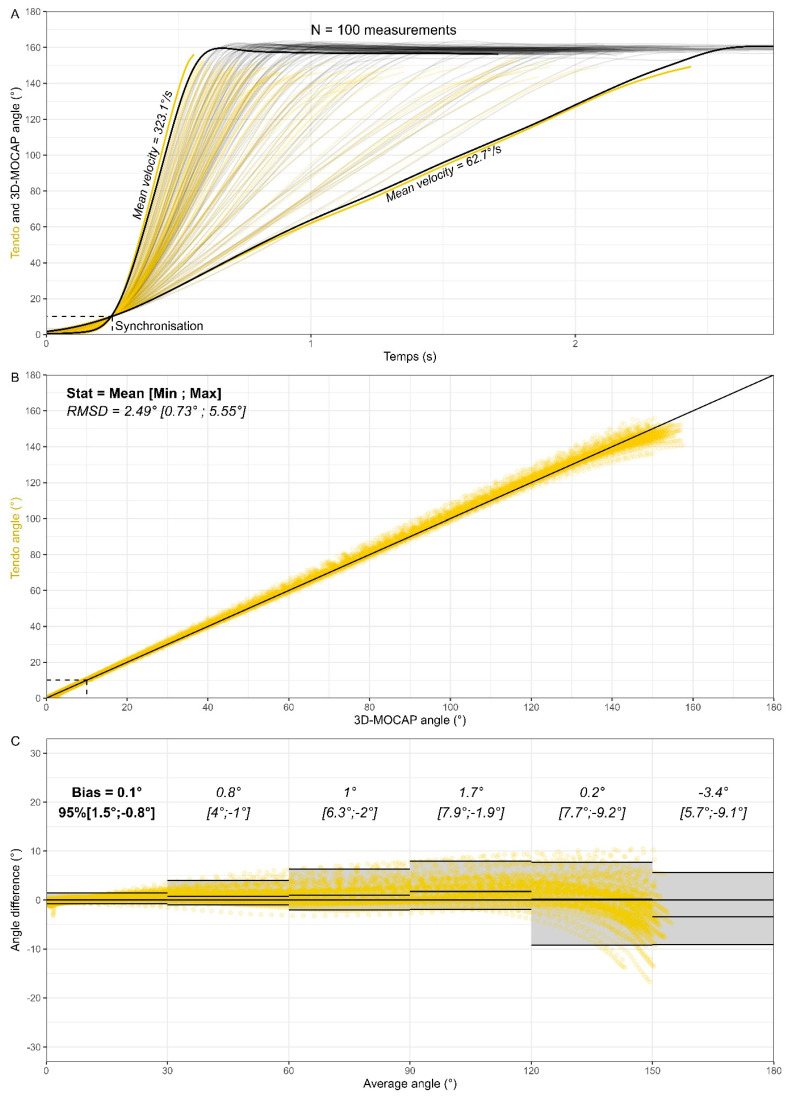
Validity and reliability of the Tendo Sport LPT to assess angular motion in the shoulder flexion protocol. Angle comparison (**A**), angle correlation (**B**) and Bland–Altman plot divided into meaningful sections (**C**). 3D-MOCAP in black and Tendo Sport in yellow.

Finally, strong agreement between methods can be observed for average angles below 120°. The systematic errors are equal to −0.6°, 1.1°, 0.7°, −3° and the LoA ranges are equal to 2.7°, 3.2°, 4.2, 7.1° for average angles, respectively, below 30°, 60°, 90° and 120°. Above 120°, both systematic and random errors increase and become significant. The magnitude of the error can be explained by the inability of the Tendo Sport to measure large angles. As can be seen in Figure 6A, the largest angle measured by the 3D-MOCAP is 178.7 ± 0.6 (Mean ± SD), whereas the same angle computed from the Tendo Sport measure yields an average angle of 137.6° ± 1.8°. Additional information about the shortcomings of the method is provided in the discussion. The results of **part III** obtained with shoulder flexion are displayed in Figure 7. As opposed to the previous section, reliability was not sought in this section. Instead, a large range of velocities was purposely performed by the subject (average velocity ranging from 62.7°/s to 323.1°/s).

The average value of the RMSD is 2.49° with individual values within 0.73° and 5.55°. While a few curves are above the 5° criteria, most of the curves are close to the gold standard, revealing a high validity of the method. Once again, strong agreement is observed between methods for average angles below 120°. The systematic errors are equal to 0.1°, 0.8°, 1°, 1.7° and the LoA range are equal to 2.3°, 5°, 8.3°, 9.8° for average angles, respectively, below 30°, 60°, 90° and 120°. The right part of the chart (i.e., large angle) still presents a larger dispersion (random error) even if the discrepancies between maximal angles are smaller than with the bike wheel (160.6° ± 1.6° for 3D-MOCAP and 147.1° ± 4.1° for Tendo Sport).

## 4. Discussion

The objective of this article was to investigate the reliability and validity of the Tendo Sport LPT as well as to explore the possibility of retrieving angular kinematics.

### 4.1. Part I

The Tendo Sport measures both time as well as distance during motion, allowing computation of velocity as well as acceleration, force and power. Error could, therefore, come from both time and/or distance measurement. According to the type of data exported by Tendo Software (i.e., fixed distance with corresponding time), it is likely that the Tendo Sport sensor uses a light source and a photo detector on each side of a rotating perforated disk. While the linear distance between the first and the second light impulse (light passing through the perforated disk) is standardized and should correspond to exactly 1 cm, the true linear distance traveled before the first light impulse is unlikely to be as accurate, since the initiation of the movement does not systematically start in front of a hole. In addition, the 2.7 m long Kevlar cord of the Tendo Sport is not guided around the spool and reels freely due to the spring responsible for the default tension. If the cord reels over itself too much, this could result in a diameter larger than the spool and, therefore, a fallacious relationship between angular displacement of the spool and the linear distance of the cord. This problem is known as spool over-wind.

Figure 5 presents potential errors coming exclusively from either time measure (facet A) or distance measure (Facet C). As mentioned by almost all authors, those small potential errors are later magnified by the important number of computations necessary in kinematic methods. The famous saying “Garbage in, Garbage out” stands true in this situation and perfectly illustrates the noise amplification due to error propagation (Table 2).

### 4.2. Part II and Part III

Once the validity and the reliability of the Tendo Sport were established in linear settings, the LPS was investigated in a constraint angular setting (bike wheel). As opposed to the 3D-MOCAP, the Tendo System does not capture the entire movement (Figure 6 and Figure 7). Instead, it captures solely the concentric part when the cable is pulled out of the sensor unit. As hypothesized, the LoA range theoretically containing 95% of the residuals is smaller in the wheel situation (Figure 6C) than in the human situation (Figure 7C). Those results were expected, since the bike wheel was used to reduce potential variability. Similar results were obtained for constrained squat jumps in a Smith machine, compared to unconstrained squat jump [25].

In a study assessing the concurrent validity and reliability of five common clinical goniometric devices, Kiatkulanusorn and colleagues also performed angular measurements with and without human movement involved. The Limits of Agreements of available clinical goniometric devices ranged between −4.11° and 4.04° on a standardized bench without biological variability and between −10.98° and 11.36° in the shoulder flexion condition [40].

**Figure 8:**
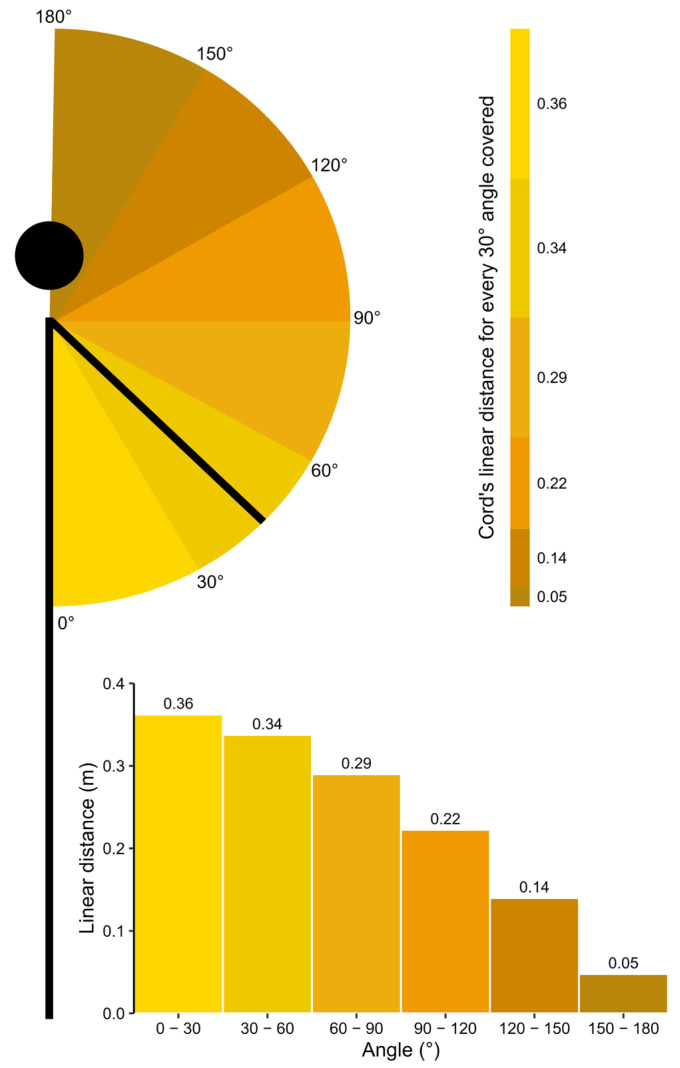
Relationship between angle covered and cord length linearly unreeled from the sensor. The length is maximal for small angles and minimal for large angles, resulting in error having a larger impact for angles greater than 120°.

Figure 8 represents the shoulder flexion simulation performed by a subject with arm length = 0.70 m. As displayed in Figure 3, the linear distance measured by an LPT at the end of the movement is equal to two-arm length (i.e., 1.40 m). The cord equal to two-arm length is presented on the right part of Figure 8 with distinct colors depending on the angle covered.

The innovative method proposed in this article seems to provide accurate and reliable results from 0° to 120° angle. Above 120° angle, both validity and reliability drop down to unsatisfactory standards. This can be explained by the geometric configuration of the method. At the beginning of the movement (0°–30°), the hand displacement is parallel to the cable unreeling due to the tripod and the pulley redirecting the cable around the initial position of the hands. Therefore, the linear displacement is maximal (0.36 m to cover 30°). At the end of the movement (150°–180°), the hand displacement is perpendicular to the cable unreeling. Therefore, the linear displacement is very small (0.05 m to cover 30°). A potential error of 0.025 m, contained within the LoA range computed from part I, represents 50% of the linear distance travel in the last 30°, whereas it represents only 6.9% in the first 30°; therefore, having a much larger impact at the end of the movement compared to the beginning.

It is also important to highlight that the humerus is not tightly fixed to the trunk but rather freely connected to the scapula. The shoulder is one of the articulations with the largest degrees of freedom, since it is designed for prehension purposes. The greater mobility of the upper limb is obtained through small and shallow articular surfaces of the glenohumeral joint in addition to the scapula being a floating bone over the posterior surface of the rib cage. In addition, care must be placed on not moving the other joints of the segment. In the present article, despite the best intention of the subject to keep his arms straight throughout the movement, the 3D-MOCAP allows quantifying angle variation between segments. Over 100 trials, the elbow angle decreased on average by 8.0° ± 1.2° between the beginning and the end of the shoulder flexion, resulting in a slight elbow flexion. While the shoulder angle computed with the 3D-MOCAP is still accurate, the angle computed with the Tendo Sport could be negatively impacted, since it could affect the length of the segment (see Figure 3).

In conjunction with the previous paragraphs, it is hypothesized that the current results could be improved with knees [41] or elbows [42] flexion/extension for multiple reasons. First, the length of the segment cannot change during the movement, since there are no additional articulations between the extremity of the limb and the joint investigated. Second, both have synovial joints that allow fewer degrees of freedom (i.e., hinge and modified hinge, respectively, for elbow and knee joints). Third, due to soft tissues being squeezed between bones, the active ranges of motion are restricted and, therefore, smaller than the shoulder joint.

Lastly, based on the data from part III, the correlation between the average velocity of the movement and the RMSD of each curve indicates a moderate correlation (R = 0.45, *p*-value = $2.645\times 10^{-6}$ 95% [0.28; 0.59]), suggesting that the validity is not affected by the velocity of the movement.

### 4.3. Limits

To the authors’ knowledge, this study is the first one to investigate the possibility of retrieving angular kinematics with an LPT. Nevertheless, some limitations need to be disclosed.

First, while gravity represents a standardized effect on the 10 kg weight, the release was performed by a human being, potentially adding a little bit of biological variability. It is highly likely that the four curves removed from part I, due to their consequent differences with the rest of the measurements, were due to the releasing technique of the weight. While the weight was guided vertically thanks to a thin metal bar inside, no technical solution prevented potential rotation of the disk around the vertical bar, potentially slightly increasing the linear distance measured by the cord in the case of rotation of the disk.

Second, the present study includes only one subject, which reduces the generalizability of its results. Due to the cumulative nature of science, additional research with more subjects will be needed to reinterpret, extend or correct the results of the present study.

## 5. Conclusions

Linear Position Transducers could be used as a surrogate tool to compute angular displacement in certain conditions. It is essential that the length of the limb does not change during the movement and that the measured distance corresponds to the distance relative to the original position of the limb. The present study deemed acceptable the validity, reliability and agreement for angular kinematics estimation with a Linear Position Transducer for angles below 120°, mostly due to the geometric configuration of the test.

## Figures and Tables

**Table 1:** Marker positions.

	Markers	Positions
**Torso**	C7 Vertebra	On the spinous process of the 7th cervical vertebra
	T10 Vertebra	On the spinous process of the 10th thoracic vertebra
	Clavicle	On the jugular notch where the clavicles meet the sternum
	Sternum	On the xiphoid process of the sternum
	Right scapula	Anywhere over the right scapula (Not included in the Plug-in Gait model calculations. No equivalent on the left side. Asymmetry helps distinguish right from left during labeling)
**Upper limb**	Shoulder (X2)	On the acromioclavicular joint
	Upper arm (X2)	On the upper lateral 1/3 surface of the arm
	Lateral elbow (X2)	On the lateral epicondyle approximating the elbow joint axis
	Medial elbow (X2)	On the medial epicondyle approximating the elbow joint axis
	Forearm (X2)	On the lower lateral 1/3 surface of the forearm
	Lateral wrist (X2)	On the lateral side of the wrist (Thumbs) as close to the wrist joint center as possible.
	Medial wrist (X2)	On the medial side of the wrist (pinky finger), as close to the wrist joint center as possible.
	Finger (X2)	On the proximal portion of the first phalanges of the index finger
**Pelvis**	Anterior (X2)	Anterior superior iliac spine
	Posterior (X2)	Posterior superior iliac spine (immediately below the sacroiliac joints, where the spine joins the pelvis)

**Table 2:** Error propagation highlighting why Linear Velocity Transducers could be more accurate than Linear Position Transducers [28] by reducing the number of computations necessary to reach key information, provided that the initial measures are accurate.

	Variable	Error
**Measure of time (T)**	Time	δTime
**Measure of distance (D)**	Distance	δDistance
**Measure of mass (M)**	Mass	δMass
**Velocity computation (V)** **First differentiation**	$Velocity=\frac{D_{n+1}-D_{n}}{T_{n+1}-T_{n}}$	$\delta Velocity=\sqrt{{\left(\frac{\sqrt{{\left(\delta D\right)}^{2}+{\left(\delta D\right)}^{2}}}{D}\right)}^{2}+{\left(\frac{\sqrt{{\left(\delta T\right)}^{2}+{\left(\delta T\right)}^{2}}}{T}\right)}^{2}}$
**Acceleration computation (A)** **Second differentiation**	$Acceleration=\frac{V_{n+1}-V_{n}}{T_{n+1}-T_{n}}$	$\delta Acceleration=\sqrt{{\left(\frac{\sqrt{{\left(\delta V\right)}^{2}+{\left(\delta V\right)}^{2}}}{V}\right)}^{2}+{\left(\frac{\sqrt{{\left(\delta T\right)}^{2}+{\left(\delta T\right)}^{2}}}{T}\right)}^{2}}$
**Force computation (F)**	Force=M×A	$\delta Force=\sqrt{{\left(\frac{\delta M}{M}\right)}^{2}+{\left(\frac{\delta A}{A}\right)}^{2}}$
**Power computation (P)**	Power=F×V	$\delta Force=\sqrt{{\left(\frac{\delta F}{F}\right)}^{2}+{\left(\frac{\delta V}{V}\right)}^{2}}$

## Data Availability

The original contributions presented in the study are included in the article, further inquiries can be directed to the corresponding author.

## Abbreviations

The following abbreviations are used in this manuscript:

1RM	One repetition with maximal amount of weight
ICC	Intraclass correlation coefficient
IMU	Inertial measurement unit
LoA	Limits of agreement
LPT	Linear position transducer
RMSD	Root mean square differences
